# Evaluation of electroencephalography biomarkers for Angelman syndrome during overnight sleep

**DOI:** 10.1002/aur.2709

**Published:** 2022-03-19

**Authors:** Yuval Levin, Nishitha S. Hosamane, Taylor E. McNair, Shrujana S. Kunnam, Benjamin D. Philpot, Zheng Fan, Michael S. Sidorov

**Affiliations:** ^1^ Center for Neuroscience Research Children's National Medical Center Washington District of Columbia USA; ^2^ The George Washington University School of Medicine and Health Sciences Washington District of Columbia USA; ^3^ Department of Cell Biology & Physiology Carolina Institute for Developmental Disabilities, and UNC Neuroscience Center, University of North Carolina Chapel Hill North Carolina USA; ^4^ Department of Neurology University of North Carolina Chapel Hill North Carolina USA; ^5^ Departments of Pediatrics and Pharmacology & Physiology The George Washington University School of Medicine and Health Sciences Washington District of Columbia USA

**Keywords:** Angelman syndrome, biomarker, delta, EEG, sleep, spindle

## Abstract

Angelman syndrome (AS) is a neurodevelopmental disorder caused by loss‐of‐function mutations in the maternal copy of the *UBE3A* gene. AS is characterized by intellectual disability, impaired speech and motor skills, epilepsy, and sleep disruptions. Multiple treatment strategies to re‐express functional neuronal *UBE3A* from the dormant paternal allele were successful in rodent models of AS and have now moved to early phase clinical trials in children. Developing reliable and objective AS biomarkers is essential to guide the design and execution of current and future clinical trials. Our prior work quantified short daytime electroencephalograms (EEGs) to define promising biomarkers for AS. Here, we asked whether overnight sleep is better suited to detect AS EEG biomarkers. We retrospectively analyzed EEGs from 12 overnight sleep studies from individuals with AS with age and sex‐matched Down syndrome and neurotypical controls, focusing on low frequency (2–4 Hz) delta rhythms and sleep spindles. Delta EEG rhythms were increased in individuals with AS during all stages of overnight sleep, but overnight sleep did not provide additional benefit over wake in the ability to detect increased delta. Abnormal sleep spindles were not reliably detected in EEGs from individuals with AS during overnight sleep, suggesting that delta rhythms represent a more reliable biomarker. Overall, we conclude that periods of wakefulness are sufficient, and perhaps ideal, to quantify delta EEG rhythms for use as AS biomarkers.

**Lay Summary:**

Electroencephalography (EEG) is a safe and reliable way of measuring abnormal brain activity in Angelman syndrome. We found that low‐frequency “delta” EEG rhythms are increased in individuals with Angelman syndrome during all stages of overnight sleep. Delta rhythms can be used as a tool to measure improvement in future clinical trials.

## INTRODUCTION

Angelman syndrome (AS) is a rare (~1:15,000) neurodevelopmental disorder characterized by intellectual disability, seizures, lack of speech, motor dysfunction, microcephaly, and sleep disturbances (Bird, 2014). Impaired sleep ‐ including the inability to fall asleep, stay asleep, and fall back asleep after waking ‐ affects up to 90% of individuals with AS, and is a common quality of life concern for affected individuals and caregivers (Thibert et al., 2013; Wheeler et al., 2017). AS is a single‐gene disorder caused by loss of expression of maternal *UBE3A* (Kishino et al., 1997). In neurons, the paternal copy of *UBE3A* is epigenetically silenced by a long noncoding antisense transcript (*UBE3A‐ATS*) (Meng et al., 2012). In mouse models of AS, multiple approaches to “unsilence” the paternal *Ube3a* allele have resulted in neuronal Ube3a protein expression and durable behavioral improvement (Huang et al., 2011; Meng et al., 2015; Milazzo et al., 2021; Schmid et al., 2021; Wolter et al., 2020). Promising clinical trials are currently underway using one such approach: an antisense oligonucleotide designed to inhibit the *UBE3A‐ATS* and unsilence paternal *UBE3A* (Elgersma & Sonzogni, 2021; Markati et al., 2021). In order for current and future trials to be successful, biomarkers are needed to assess target engagement and/or the effectiveness of treatments. Ideal biomarkers for AS clinical trials should be safe, easily obtained, quantifiable, robust, biologically based, and linked to meaningful AS phenotypes (Jeste et al., 2015).

Low‐frequency delta (~2–4 Hz) EEG rhythms represent a promising AS biomarker that meets each of the above criteria. First, EEG is a safe and noninvasive tool to measure patterned neural activity that is well‐tolerated in children with neurodevelopmental disorders including AS (Wang et al., 2013). Increased delta rhythms are present in >80% of individuals with AS (Vendrame et al., 2012), and are also seen in rodent models of AS (Born et al., 2017; Born et al., 2021; Copping & Silverman, 2021; Sidorov et al., 2017). Increased delta rhythms are quantifiable and robust in AS: they are present across the brain, during periods of both wake and sleep, and across childhood development (Frohlich et al., 2019; Sidorov et al., 2017). In individuals with AS, the strength of delta rhythms correlates with symptom severity across multiple domains, including cognitive, motor, communication, and severity of epilepsy (Hipp et al., 2021; Ostrowski et al., 2021). For these reasons, delta power represents a promising biomarker for assessing target engagement and for tracking improvement in AS in response to treatment. Optimizing the conditions for reliably measuring and comparing delta power to other putative EEG biomarkers in AS will help to guide the design of clinical trials.

Given the high prevalence of sleep impairments in AS and the well‐established relationship between delta rhythms and slow‐wave sleep (Hirshkowitz, 2004), we asked whether delta rhythms can be more reliably measured in sleep versus wake. While recent work correlated the strength of delta rhythms with AS clinical severity across many domains (Hipp et al., 2021; Ostrowski et al., 2021), these studies did not assess the relationship between delta power and impaired sleep. Our prior work (Sidorov et al., 2017) found increased delta during sleep in AS; however, a limitation of this study was the duration and quality of sleep used. There, we analyzed short daytime EEGs, where only ~50% of EEGs included periods of sleep and the average length of sleep was ~20 min. Here, we quantified delta rhythms in EEGs obtained during overnight polysomnography, averaging ~5–7 h of sleep. We compared AS EEGs to EEGs from age and sex‐matched neurotypical individuals and individuals with Down syndrome. In addition, we assessed sleep spindles during overnight EEGs, as prior work using daytime EEGs suggested that spindles may be impaired during short periods of sleep (den Bakker et al., 2018).

We report that delta rhythms were reliably increased in AS during wake and during all stages of NREM sleep in overnight EEGs. However, the degree to which delta was increased in AS relative to NT controls was not greater in sleep than during wake. In addition, sleep spindle impairments were difficult to detect via EEG measurements taken during overnight sleep. Overall, we conclude that overnight sleep EEG does not confer additional benefit relative to wake EEG for the detection of AS EEG biomarkers.

## METHODS

### Subjects and data acquisition

**FIGURE 1:**
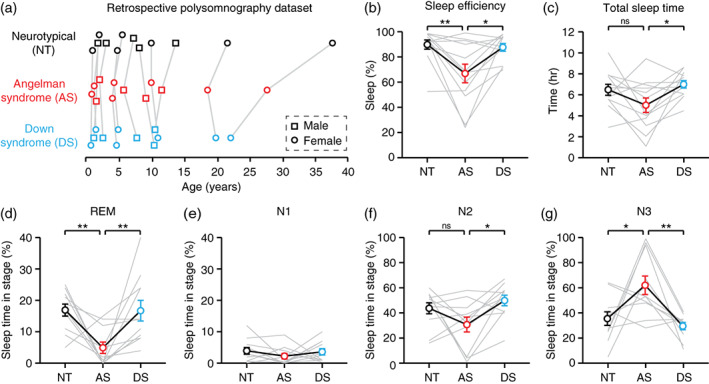
Overnight sleep is impaired in individuals with Angelman syndrome. (a) Age and sex matching of overnight polysomnography data (*n* = 12 per genotype). NT, neurotypical; AS, Angelman syndrome; DS, down syndrome. (b) Sleep efficiency was decreased in AS. (c) Total sleep time was decreased in AS relative to DS, but not NT. (d–g) percentage of sleep spent in each sleep stage: (d) REM, (e) N1, (f) N2, (g) N3. *p <0.05, **p <0.01 reflect post hoc tests. Error bars indicate ± SEM

We analyzed retrospective, de‐identified inpatient polysomnography data acquired by the UNC Sleep Disorders Center from 2006 to 2020. Overnight EEGs from individuals with a confirmed AS diagnosis were age and sex matched with EEGs from neurotypical (NT) individuals and individuals with Down syndrome (DS) (Figure 1a). Subjects of all three genotypes were referred for overnight polysomnography because of obstructive sleep apnea (Figure S1A). We analyzed 12 EEGs per genotype (5 male, 7 female). Subjects ranged in age from 10 months to 37 years, averaging 8.7 ± 1.4 years. Wake and sleep EEG data were collected from the same subjects within sessions. Sleep periods analyzed during EEG analysis (5.3 ± 0.3 h) were significantly longer than wake periods analyzed during EEG analysis (1.7 ± 0.2 h) (Figure S5A). AS polysomnography included a single EEG from five subjects, two EEGs from two subjects, and three EEGs from one subject. In cases where multiple EEGs were analyzed from the same subject, the time between EEGs ranged from 5 months to 9 years. Each NT and DS EEG was from a unique subject. Molecular diagnoses of individuals with AS included one subject (one EEG) with a Class I deletion, two subjects (three EEGs) with a Class II deletion, four subjects (seven EEGs) with a deletion but class not specified, and one subject (one EEG) with uniparental disomy. Multichannel polysomnograms were recorded digitally and stored using Natus Systems or Grass Systems hardware and software. Sleep staging was determined by trained technicians and physicians board‐certified in sleep medicine with access to video. EEGs were acquired using sampling rates of 200 or 256 Hz using six electrodes: two frontal channels (F3, F4), two central channels (C3, C4), and two occipital channels (O1, O2).

### Sleep analysis

Technician and physician‐defined sleep staging data were used to analyze sleep quality and sleep architecture (Figure 1, Figure S1). Sleep efficiency was defined as the percentage of time spent asleep beginning at initial sleep onset and ending on final waking at the end of the session. The percentage of time spent in each sleep stage represents percentage of total sleep time spent in that stage, with periods of wake excluded. First REM latency was defined as the time from sleep onset until the beginning of the first period of REM sleep. Apnea/hypopnea index (AHI), average O_2_ saturation, percent time under 88% O_2_ saturation, and body mass index (BMI) data were provided in sleep reports. One AS EEG was excluded from sleep architecture analysis because it was coded for entirely N1 sleep (technician was not able to determine sleep stage accurately due to high background delta). The NT and DS studies matched to this file were also excluded from sleep analysis. Thus *n* = 12 per group for analyses of total sleep time and sleep efficiency, but *n* = 11 per group for sleep stage analyses. BMI and percent time under 88% O_2_ saturation data were not available for all subjects.

### EEG preprocessing

EEGs were preprocessed in MATLAB using a similar pipeline to previous work (den Bakker et al., 2018; Sidorov et al., 2017). First, data were exported from native software to .edf or .csv file formats using a referential montage and including 0.1 Hz high‐pass, 70 Hz low‐pass, and 60 Hz band‐stop filtering. Raw data were then re‐referenced to linked ears ((A1 + A2)/2) and underwent additional 1 Hz high‐pass filtering. Timestamps marked by technician staging identified boundaries for periods of wake and sleep, or sleep stages. Artifacts were manually identified and excluded, and noisy or absent channels were excluded from analysis. In sum, 16 channels were excluded from analysis (of 216 total). Channels were excluded from three NT EEGs, five AS EEGs, and two DS EEGs. There were no group differences between the amount of data excluded due to artifacts during either wake (*F*_(2,22)_ = 1.776, *p* = 0.1927) or sleep (*F*_(2,22)_ = 0.7103, *p* = 0.8027) periods of EEG. All REM sleep was excluded, and spectral analyses represent either wake, NREM sleep, or individual NREM sleep stages (N1, N2, and N3) as noted. For EEG analyses during individual sleep stages, subjects were excluded if less than 2 min of data were available during a stage.

### Spectral analysis

**FIGURE 2:**
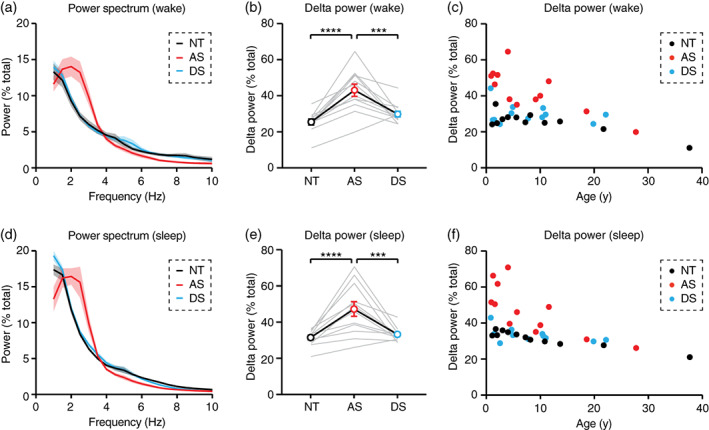
Delta power is increased in Angelman syndrome EEGs during wake and sleep. NT, neurotypical; AS, Angelman syndrome; DS, down syndrome. (a–c) Delta power is increased in AS during wake. (a) Power spectrum of group data (*n* = 12 per group); shaded area indicates ± SEM. (b) Delta power (2–4 Hz) is increased during wake in AS relative to NT and DS controls. (c) Enhanced delta power in AS during wake is most prominent early in development. (d–f) Delta power is increased in AS during NREM sleep. (d) Power spectrum of group data (*n* = 12 per group). (e) Delta power is increased during sleep in AS relative to NT and DS controls. (f) Enhanced delta power in AS during sleep is most prominent early in development. ***p <0.001, ****p <0.0001 reflect post hoc tests. Error bars indicate ± SEM

**FIGURE 3:**
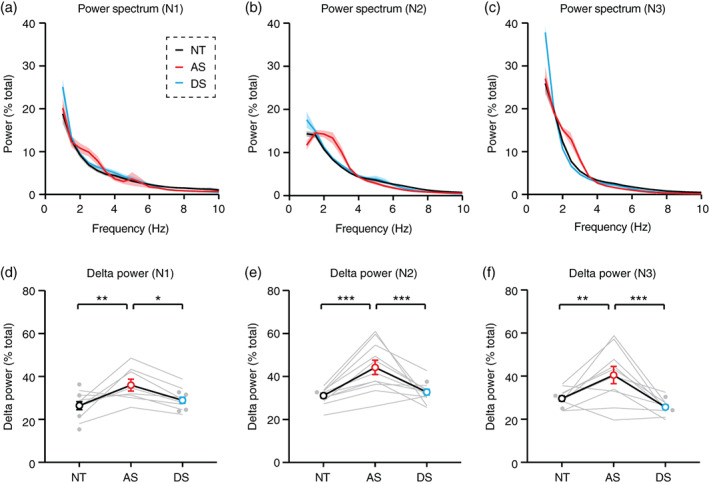
Delta power is increased in individuals with Angelman syndrome during all phases of non‐REM sleep. NT, neurotypical; AS, Angelman syndrome; DS, down syndrome. (a–c) Averaged power spectra during (a) N1, (b) N2, and (c) N3. Shaded area indicates ± SEM. (d–f) Delta power is specifically increased in AS during (d) N1 (NT: *n* = 12, AS: *n* = 8, DS: *n* = 12), (e) N2 (NT: *n* = 12, AS: *n* = 11, DS: *n* = 12), and (f) N3 (NT: *n* = 12, AS: *n* = 10, DS: *n* = 12). *p <0.05, **p <0.01, ***p <0.001 reflect post hoc tests. Error bars indicate ± SEM

We performed spectral analysis of preprocessed wake and sleep EEG data using methods identical to our prior study (Sidorov et al., 2017). Briefly, we performed fast Fourier analysis on each channel in MATLAB using the spectrogram() function with a time bin of 2 s and a 1 s overlap between bins. Relative spectral power was defined as power within a bin of interest divided by the total power between 1 and 50 Hz. Relative power was averaged across all time bins to generate power spectra and analyze delta, defined as relative power in the 2–4 Hz band. We analyzed delta power across all six electrodes in periods of wake (Figure 2a–c), sleep (Figure 2d–f), and individual NREM sleep stages (Figure 3). In addition, we compared delta power regionally during sleep and wake (Figure S2) using frontal (F3, F4), central (C3, C4), and occipital (O1, O2) groupings. Delta was defined as 2–4 Hz for all analysis as in previous work (Sidorov et al., 2017) except in Figure S3, where the bandwidth was extended to 1–4 Hz. The purpose of Figure S3 was to confirm that delta is increased in stage N3 of sleep in all groups.

### Spindle detection

We examined the quantity, duration, and peak frequency of sleep spindles (Figure 5) using two spindle detection algorithms. First, we used the same detector (Kim et al., 2015) that we previously used to quantify spindles during periods of sleep in short daytime AS EEGs (den Bakker et al., 2018). Our prior work confirmed that spindle detection using this automated approach was largely consistent with detection performed by two independent clinical experts (den Bakker et al., 2018). Briefly, raw NREM sleep EEG data were band‐pass filtered between 11 and 16 Hz and Hilbert transformed to calculate the instantaneous amplitude of the signal. If the instantaneous amplitude exceeded 5.5 times the baseline signal amplitude, a spindle was detected. The start and end of the spindle was defined by when the instantaneous amplitude crossed 2.5 times the mean signal amplitude. Spindles were included in analysis if their duration was between 0.4 and 2.0 s. If spindles were detected across multiple channels and their initiation differed by <300 ms, they were considered to be a single spindle. Spindle quantity (Figure 5d, Figure S6A,D) was defined as the number of spindles detected across all channels divided by the time of the recording. Spindle duration (Figure 5e, Figure S6B,E) was defined as the average length of all spindles detected within a session across all channels. To ensure that the same number of channels were compared across trios, if a channel was excluded from any EEG analysis due to noise, then it was also excluded from paired EEGs for the purposes of spindle analyses. We quantified the peak frequency of spindles (Figure 5F, Figure S6C,F) by performing spectral analysis of the raw data where spindles were detected and calculating the local maximum of spectral power between 11 and 16 Hz, rounded to the nearest 0.5 Hz. Peak frequency and duration were averaged across all spindles detected per EEG session, and “n” represents the number of sessions. As noted, spindles were analyzed separately during all NREM sleep, the first 20 min of NREM sleep (to compare results to den Bakker et al., 2018), and all N2 sleep.

Additional spindle detection (Figure 5g–i) was performed using yet another spindle algorithm (YASA) (Vallat & Walker, 2021), a Python‐based open‐source spindle detector, using Jupyter Lab. First, YASA band‐pass filters raw EEG data between 1 and 30 Hz, resulting in the filtered signal EEG_*bf*_. YASA also band‐pass filters the raw EEG data between 12 and 15 Hz (~11–16 Hz, including filter roll‐off) to create a sigma‐filtered signal, EEG_σ_. YASA then calculates the relative sigma power, root mean square, and moving correlation between EEG_*bf*_ and EEG_σ_. In order for a spindle to be detected by YASA, each of these three parameters must eclipse a threshold in the same time window: (a) relative sigma power ≥0.2, (b) root mean square ≥ RMS_mean_ + 1.5 * RMS_std_, and (c) moving correlation ≥0.65. If all three thresholds are crossed, a potential spindle is detected. The start and end of the spindle is determined by when two of the three thresholds are crossed, and potential spindles between 0.5 and 2.0 s are considered spindles. Spindles detected within 500 ms of one another on the same channel were merged and considered a single spindle. YASA also calculates the median instantaneous frequency of each spindle using a Hilbert transform. YASA does not automatically remove spindles detected across multiple channels, so we removed duplicates post hoc whose initiation differed by <1 s.

### Statistical analysis

**FIGURE 4:**
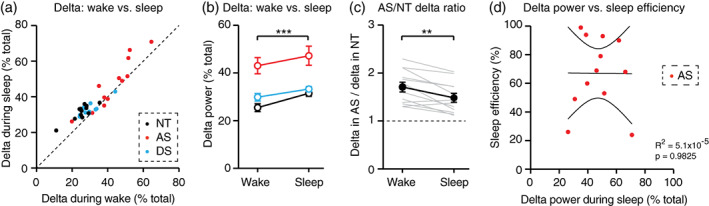
Increased delta power in Angelman syndrome is more pronounced during wake than during sleep. NT, neurotypical; AS, Angelman syndrome; DS, down syndrome. (a) Delta power during wake versus delta power during NREM sleep. Each dot represents one overnight EEG that included both periods of wake and sleep. Dotted line indicates wake = sleep; points above the line (83%) indicate sessions where delta is higher in sleep than in wake. (b) Quantification of delta in wake and sleep within individuals reveals that delta power is higher in sleep across genotypes. (c) Between AS‐NT pairs, increases in delta power (“AS/NT delta ratio”) are greater during wake than during sleep. (d) Delta power during NREM sleep is not correlated with sleep efficiency in individuals with AS. Overlaid lines in represent best‐fit ±95% confidence interval. **p <0.01, ***p <0.001. Error bars indicate ± SEM

**FIGURE 5:**
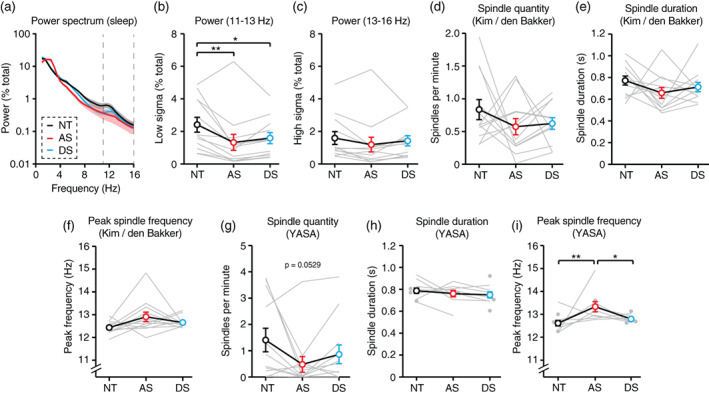
Sleep spindle impairments in Angelman syndrome are difficult to detect during overnight sleep. NT, neurotypical; AS, Angelman syndrome; DS, down syndrome. (a) Power spectrum of group data (*n* = 12 per group). Dotted lines indicated sigma (11–16 Hz) band where spindles occur; shaded area indicates ± SEM. (b) Spectral power is decreased in AS from 11–13 Hz. (c) Spectral power is normal in AS from 13–16 Hz. (d) Spindle quantity, (e) spindle duration, and (f) peak spindle frequency during NREM sleep, as detected by Kim/den Bakker detector (*n* = 12 per group). (g) Spindle quantity (*n* = 12 per group), (h) spindle duration (NT: *N* = 12, AS: *N* = 9, DS: *N* = 12), and (i) peak spindle frequency (NT: *N* = 12, AS: *N* = 9, DS: *N* = 12) during NREM sleep, as detected by YASA. *p <0.05, **p <0.01 reflect post hoc tests. Error bars indicate ± SEM

A one‐way repeated measures (RM) ANOVA was used for most comparisons of sleep (Figure 1b–g, Figure S1A,B,F,G), delta power (Figure 2b,e, Figures S2A,B,D,E, S3A,C), and spindles (Figure 5b–g, Figure S6D) between matched NT, AS, and DS data. In cases where data points were absent or excluded, a mixed‐effects analysis was used (Figures S1C–E and S2C,F, Figure 3d–f, Figure S3B, Figures 4d and 5h,i, and Figures S5E and S6A–C,E‐F). Where a statistically significant main effect was found, a Tukey–Kramer post hoc test was used to make direct comparisons between individual groups. We used a two‐way ANOVA with age and genotype as factors, and two continuous measures (age and delta power), to assess the effects of age and genotype on delta power (Figure 2c,f). We used a two‐way RM ANOVA for Figure 4b and Figure S5A,C. We used a two‐way RM ANOVA, with time and genotype as factors, for Figure S5A. Simple linear regression was used to assess relationships between delta power in different sleep stages within sessions (Figure S4), between delta and sleep efficiency (Figure 4d), and between spindle quantity and AHI, average O_2_ saturation, and age (Figure S7). A paired t‐test was used in Figure 4c to compare the AS/NT delta ratio during wake and sleep within individuals, and in Figure S5D to compare the AS/NT delta ratio across sleep within individuals. Statistical analysis was performed in Prism 9 and JMP 16. All data are plotted as mean ± SEM and *p <0.05, **p <0.01, ***p <0.001, ****p <0.0001.

## RESULTS

### Overnight sleep is impaired in individuals with Angelman syndrome

We compared sleep quality and sleep architecture in individuals with AS to age and sex‐matched neurotypical (NT) and Down syndrome (DS) controls, with subjects ranging in age from 10 months to 37 years (Figure 1a). Subjects were referred for polysomnography because of sleep apnea, but the severity of sleep apnea, and the body mass index of subjects, did not differ by genotype (Figure S1A–D). Sleep efficiency, defined as the percentage of time spent asleep after initial sleep onset, was decreased in AS relative to NT and DS controls (Figure 1b; one‐way RM ANOVA, *F*_(2,22)_ = 7.674, *p* = 0.0030; post hoc NT‐AS: *p* = 0.0049; AS‐DS: *p* = 0.0107). Total sleep time was significantly decreased in AS relative to DS, but not relative to NT (Figure 1c; *F*_(2,22)_ = 3.856, *p* = 0.0367; post hoc NT‐AS: *p* = 0.1391; AS‐DS: *p* = 0.0356). Individuals with AS spent significantly less time in REM sleep (Figure 1d; *F*_(2,20)_ = 7.890, *p* = 0.0030; post hoc NT‐AS: *p* = 0.0066; AS‐DS: *p* = 0.0074), with an increased latency to the first bout of REM sleep (Figure S1E). The percentage of sleep spent in stage N1 did not differ between groups (Figure 1e; *F*_(2,20)_ = 0.7180, *p* = 0.4999). The percentage of sleep spent in stage N2 was decreased in AS relative to DS, but not relative to NT (Figure 1f: *F*_(2,20)_ = 7.890, *p* = 0.0268; post hoc NT‐AS: *p* = 0.1479; AS‐DS: *p* = 0.0233). The percentage of sleep spent in stage N3 was increased in AS relative to NT and DS controls (Figure 1g; *F*_(2,20)_ = 8.009, *p* = 0.0028; post hoc NT‐AS: *p* = 0.0158; AS‐DS: *p* = 0.0034). The amount of stage shifts and wakings were not different between genotypes (Figure S1F,G). Overall, our results are consistent with prior reports of impaired sleep in AS (Miano et al., 2004), confirming that our sample is representative of typical AS sleep.

### Delta power is increased during wake and all stages of non‐REM sleep in Angelman syndrome

During periods of wakefulness, 2–4 Hz delta power was increased in AS relative to NT controls (Figure 2a,b; *F*_(2,22)_ = 16.84, *p* <0.0001; post hoc NT‐AS: *p* <0.0001), and increased delta power was specific to AS (post hoc AS‐DS: *p* = 0.0003). Increased delta power in AS during wake was age‐dependent (Figure 2c; age X genotype interaction: *F*_(5,30)_ = 6.3938, *p =* 0.0049). During NREM sleep, delta power was increased in AS relative to NT controls (Figure 2d,e; *F*_(2,22)_ = 16.52, *p* <0.0001; post hoc NT‐AS: *p* <0.0001), and increased delta power was specific to AS (post hoc AS‐DS: *p* = 0.0003). Increased delta power in AS during NREM sleep was age‐dependent (Figure 2f; age X genotype interaction: *F*_(5,30)_ = 6.6976, *p =* 0.0039). During both wake and NREM sleep, delta power was increased in AS in frontal, central, and occipital electrodes (Figure S2).

To compare delta power across sleep, we calculated power spectra separately during each NREM sleep stage: N1, N2, and N3 (Figure 3a–c). Delta power was increased in AS relative to NT during N1 (Figure 3d; *F*_(2,18)_ = 7.271, *p* = 0.0048; post hoc NT‐AS: *p* = 0.0038), N2 (Figure 3e; *F*_(2,21)_ = 15.15, *p* <0.0001; post hoc NT‐AS: *p* = 0.0001), and N3 (Figure 3f; *F*_(2,20)_ = 13.72, *p* = 0.0002; post hoc NT‐AS: *p* = 0.0034). Increases in delta power were specific to AS in each sleep stage (Figure 3d–f; post hoc AS‐DS for N1: *p* = 0.0318, for N2: *p* = 0.0006, for N3: *p* = 0.0001). Delta power was greater during N3 than N1 and N2 in all groups (Figure S3), confirming that N3 staging was generally accurate. Within individuals, delta power was strongly correlated between sleep stages (Figure S4). Overall, increased delta power was seen in AS EEGs across wake, across all stages of NREM sleep, and across all electrodes.

### Increased delta power in Angelman syndrome relative to neurotypical controls is more pronounced during wake than during sleep

The primary goal of this study was to determine whether wake EEGs or sleep EEGs are best suited to detect increased delta power as an AS biomarker. To directly address this question, we compared delta power during wake and NREM sleep within individual overnight EEGs. As expected, delta power was higher during sleep periods in the majority (30/36) of total EEGs (Figure 4a). The increase in delta rhythms during sleep relative to wake was statistically significant (Figure 4b; RM two‐way ANOVA: main effect of sleep/wake state: *F*_(1,11)_ = 29.95, *p* = 0.0002). Post‐hoc tests revealed a statistically significant increase in delta power during sleep relative to wake in NT EEGs (*p* <0.0001) and DS EEGs (*p* = 0.0084) and a trend toward increased delta power in AS EEGs (*p* = 0.0624).

To determine whether wake or sleep is better suited for detecting increased delta as an AS biomarker, we compared delta power between AS‐NT matched pairs during wake and during sleep. Here, we restricted analysis to AS and NT EEGs as this direct comparison is most relevant from a biomarker perspective. We calculated an “AS/NT delta ratio,” defined as delta power in an AS EEG divided by delta power in the matched NT EEG. The AS/NT delta ratio serves as a measure of the degree to which delta power is increased in AS. During both wake and sleep, the AS/NT delta ratio was greater than 1, confirming that increased delta can be detected during both states (Figure 4c). Interestingly, the AS/NT delta ratio was significantly higher in wake than in sleep (Figure 4c; paired t‐test: *t*_(11)_ = 3.972, *p* = 0.0022). This result suggests that the relative increase in delta power in AS is greater during wake (71 ± 10% increase) than during overnight sleep (48 ± 9% increase). We confirmed that the increased AS/NT delta ratio during wake was not due to differences in recording length between wake and sleep EEGs (Figure S5A): delta power itself and the AS/NT delta ratio remained consistent across the duration of NREM sleep (Figure S5B–D). We then asked whether restricting EEG analysis to a single sleep stage might improve detection of increased delta in individuals with AS. The AS/NT delta ratio was not different between stages N1, N2, and N3 (Figure S5E). Thus, we conclude that while increased delta rhythms in AS can be detected reliably during EEGs from overnight sleep, overnight sleep does not provide additional benefit over periods of wake for measurements of the delta EEG phenotype.

Although sleep EEG does not provide an advantage in detecting increased delta power in AS, one potential advantage of sleep EEG is the ability to correlate delta with sleep quality within individuals. However, there was no correlation between delta power during NREM sleep and sleep efficiency in individuals with AS (Figure 4d; *R* ^2^ = 5.1 × 10^−5^, *p* = 0.9825). These results provide further evidence that sleep EEG does not provide additional benefit over wake EEG for quantifying delta as an AS EEG biomarker.

### Sleep spindle impairments are difficult to detect in Angelman syndrome EEGs

In a prior exploratory study, we found that the quantity and duration of sleep spindles during short periods of sleep during daytime EEGs were decreased in individuals with AS (den Bakker et al., 2018). Here, we attempted to replicate these findings during overnight EEGs in order to evaluate spindles as a potential AS EEG biomarker. During NREM sleep, spectral power (Figure 5a) was decreased in AS relative to NT controls in the low sigma band (11–13 Hz) (Figure 5c; *F*_(2,22)_ = 6.413, *p* = 0.0064; post hoc NT‐AS: *p* = 0.0064), but not in the high sigma band (13–16 Hz) (Figure 5d; *F*_(2,22)_ = 1.635, *p* = 0.2177). Spectral power was also decreased in DS relative to NT controls in the 11–13 Hz band (Figure 5c; post hoc NT‐DS: *p* = 0.0419). Decreased spectral power in the 11–13 Hz range is consistent with findings from our prior study and suggested that there may be a decrease in sleep spindles in AS during overnight sleep.

To quantify spindles, we first used the same automated spindle detection algorithm as in our previous study (den Bakker et al., 2018; Kim et al., 2015). Surprisingly, automated spindle detection using the Kim/den Bakker detector revealed no difference in the quantity (Figure 5d; *F*_(2,22)_ = 0.9699, *p* = 0.3948) or duration (Figure 5e; *F*_(2,22)_ = 1.502, *p* = 0.2447) of spindles in individuals with AS. The peak frequency at which spindles occurred was also not statistically different between groups, though there was a trend toward increased peak frequency in AS (Figure 5f; *F*_(2,22)_ = 2.852, *p* = 0.0792). Sleep spindles are a defining characteristic of N2 sleep (Andrillon et al., 2011); therefore, we asked whether restricting spindle detection to periods of N2 sleep is better able to detect impairments in AS. However, within N2 only, there was no difference in the amount (Figure S6A) or duration (Figure S6B) of spindles in individuals with AS. During N2 only, there was a statistically significant increase in peak spindle frequency in AS EEGs (Figure S6C). In our prior study, sleep during daytime EEG recordings averaged ~20 min in length (den Bakker et al., 2018); therefore, we also analyzed only the first 20 min of overnight EEGs to ask whether spindle impairments are present in AS only at the beginning of sleep. Contrary to our earlier findings, spindle quantity during the first 20 min of NREM sleep was not different between AS and NT groups (Figure S6D), but spindle duration was decreased in AS EEGs (Figure S6E). Peak spindle frequency was not different by group in the first 20 min of NREM sleep EEGs (Figure S6F). Overall, statistically meaningful impairments in sleep spindles in individuals with AS EEGs proved difficult to detect during overnight sleep using the Kim/den Bakker spindle detector.

We next performed a secondary spindle analysis using YASA (Vallat & Walker, 2021), a newer open‐source spindle detector that uses multiple criteria simultaneously to identify spindles (see Methods). YASA was adapted from the “A7” spindle detection algorithm that performed best among five detectors at matching human expert performance (Lacourse et al., 2019), and has been previously used to demonstrate that spindles are impaired during sleep in subjects with 15q11.2–13.1 duplication (Dup15q) syndrome (Saravanapandian et al., 2021). YASA detected a trend toward a decrease in spindles in AS EEGs (Figure 5g; *F*_(2,22)_ = 3.369, *p* = 0.0529), but no difference in spindle duration by group (Figure 5h; *F*_(2,16)_ = 0.5670, *p* = 0.5782). YASA did detect an increase in peak spindle frequency that was specific to AS EEGs (Figure 5i; *F*_(2,27)_ = 6.707, *p* = 0.0043; post hoc NT‐AS: *p* = 0.0038; post hoc AS‐DS: *p* = 0.0383). Spindles detected by YASA were positively correlated with age, but spindles detected by the Kim/den Bakker were not positively correlated with age (Figure S7A,B). Taken together, results from multiple spindle detectors and multiple conditions illustrate the difficulty of reliably detecting spindle impairments for use as biomarkers in AS.

There were a number of differences in study design that may have contributed to the difficulty in detecting spindle impairments in AS as previously reported (den Bakker et al., 2018) (see Discussion). One possibility is that the presence of obstructive sleep apnea, which is known to affect sleep spindles (Brockmann et al., 2018; Brockmann et al., 2020; Vallat & Walker, 2021), presented a confounding variable. We tested this hypothesis directly by correlating two measures of sleep‐related breathing impairment (AHI and average O_2_ saturation; Figure S1) with sleep spindles. Neither measure of sleep apnea severity correlated with spindle quantity, as measured by either the Kim/den Bakker or YASA detector (Figure S7C–F). Thus, we conclude that sleep apnea is unlikely to be the primary reason why we were unable to reliably detect spindle impairments in AS EEGs in this study.

## DISCUSSION

Increased delta rhythms have long been reported clinically in AS (Boyd et al., 1988; Thibert et al., 2013; Vendrame et al., 2012), and recent quantitative EEG studies have confirmed that increased delta power is robust and has potential as an AS biomarker (Frohlich et al., 2019; Frohlich et al., 2020; Hipp et al., 2021; Martinez et al., 2020; Ostrowski et al., 2021; Sidorov et al., 2017). In order to guide effective use of delta as a biomarker, we sought to define ideal conditions in which to measure increased delta power in AS EEGs. Delta rhythms were increased in AS during periods of wake and periods of overnight NREM sleep (Figure 2), including all stages of NREM sleep (Figure 3). Increased delta power was specific to AS (did not generalize to down syndrome controls) and was more prevalent earlier in development (Figure 2 and 3). Interestingly, the increased delta power in AS EEGs was greater during wake than during sleep (Figure 4). This result suggests that overnight sleep EEGs do not provide additional benefit over wake EEGs for detecting increased delta as an AS biomarker.

The sufficiency of wake EEGs for delta assessment provides several practical benefits. Formal overnight sleep studies often require travel and preparation, and can be challenging for children with AS and their families. In‐home overnight EEG avoids some of these challenges, but often requires a site visit for setup, continuous overnight monitoring, and introduces the risk of reduced data quality or data loss relative to a sleep lab. Short, standardized wake EEGs are well‐tolerated in individuals with AS and are ideal for use in a clinical trial setting. However, beyond detection of delta itself, overnight sleep studies do have the potential to provide additional benefit. First, overnight EEGs provide an opportunity to directly correlate delta power with sleep quality within AS individuals. Linking putative biomarkers to meaningful phenotypes is critical (Jeste et al., 2015), and impaired sleep is among the most common features of AS (Thibert et al., 2013). However, delta power did not correlate with sleep quality in individuals with AS (Figure 4d). In addition, the absence of substantial movement artifacts during sleep EEGs has the potential to improve data quality relative to wake EEGs. However, recent work demonstrated that removing movement artifacts may not be necessary to detect increased delta or link increased delta to cognitive performance in AS (Ostrowski et al., 2021). Thus, overnight sleep EEGs provide neither primary nor secondary benefit for assessing delta power in AS.

While sleep EEGs do not provide additional benefit for detecting delta, sleep itself represents a valuable AS biomarker. Sleep impairments are common in children with AS and are particularly burdensome for caregivers (Wheeler et al., 2017). Here, we demonstrate that individuals with AS have reduced sleep efficiency, reduced REM sleep, increased time spent in stage N3, and trends toward decreased overall total sleep time and time spent in stage N2 (Figure 1). All sleep impairments were specific to AS and did not generalize to DS controls. However, sleep staging was likely imperfect in this study due to strong background delta rhythms in AS and technicians not familiar with staging AS sleep specifically. In particular, the increased scoring of N3 sleep in AS EEGs may reflect challenges distinguishing increased background delta from true slow wave sleep. Despite staging challenges, our results are consistent with a prior sleep polysomnography study in AS that lacked quantitative EEG analysis (Miano et al., 2004). Miano and colleagues also reported decreased sleep efficiency, decreased REM, increased N3, decreased N2, and a trend toward decreased total sleep time in individuals with AS. In AS, the absolute percentage of time spent in each sleep stage was comparable between our study and prior work (Miano et al., 2004) (Figure 1; REM: 4.9% vs. 11.8%, N1: 2.3% vs. 3.4%, N2: 30.7% vs. 39.5%, N3: 62.0% vs. 34.7%), though increased time in N3 likely reflects staging challenges. These results confirm that our sample is representative of typical AS sleep patterns. More broadly, polysomnography during a single overnight sleep study appears sufficient to reliably detect sleep impairments in AS. Sleep itself, independent of EEG, should be considered as a potential outcome measure in AS clinical trial settings. We propose that sleep efficiency is an ideal measure for populations with AS during overnight polysomnography: it is more reliably impaired than total sleep time, and is not affected if sleep staging is imperfect.

One limit to this study is that we did not assess AS clinical severity, and thus were unable to determine whether sleep delta or wake delta better correlates with clinical severity. However, recent work demonstrated that EEG delta power during both wake (Hipp et al., 2021; Ostrowski et al., 2021) and sleep (Ostrowski et al., 2021) is correlated with clinical severity in AS. These studies used behavioral data gathered across multiple domains via standardized tests including the Bayley Scales of Infant and Toddler Development, the Vineland Adaptive Behavior Scales, and an AS‐specific AS Clinical Severity Scale (Keute et al., 2021). Together, this work demonstrated that delta EEG power is correlated with cognitive performance, receptive and expressive communication, motor skills, and earlier onset of epilepsy. Another potential limit to using delta as a biomarker may be the age‐dependence of phenotypes in AS (Vendrame et al., 2012). Our work confirms that the value of delta as an AS biomarker may wane in adulthood (Figure 2). However, our sample size beyond age 18 was quite limited (*n* = 2 per group). A comprehensive quantitative study of EEGs in adulthood in AS would be valuable to better understand the limits of delta as a biomarker. In addition, future work should explore alternative EEG biomarkers for adults with AS.

While previous studies have linked increased delta power to cognitive impairment in AS (Hipp et al., 2021; Ostrowski et al., 2021), we included an age and sex‐matched Down syndrome control group to demonstrate that sleep (Figure 1) and delta EEG power (Figure 2) impairments are specific to AS, and do not generalize to other populations with cognitive impairment. This result suggests that cognitive impairment can occur in the absence of increased delta, thus, it is unlikely that increased delta itself is a cause of cognitive impairment in AS; rather, delta appears to represent a general readout of AS severity across multiple domains.

Sleep spindles are short, ~11–16 Hz bursts of activity that are linked to memory consolidation and are impaired in a number of neurodevelopmental and neuropsychiatric disorders (Farmer et al., 2018; Ferrarelli et al., 2007; Gorgoni et al., 2020; Gruber & Wise, 2016; Saravanapandian et al., 2021; Ulrich, 2016). By band‐pass filtering signals in the range of spindles, we are able to quantify spindles in AS EEGs despite increased background delta. We previously performed an exploratory study of sleep spindles during short (~20 min) periods of sleep using daytime EEGs and found that the quantity and duration of spindles may be decreased in AS (den Bakker et al., 2018). Mild impairments in sleep spindles have also been observed in a mouse model of AS (Copping & Silverman, 2021). Surprisingly, we found no statistically significant differences in spindle quantity, spindle duration, or peak spindle frequency in AS during overnight sleep using identical preprocessing and detection methods to our prior study (Figure 5). There were a number of methodological differences that might account for these disparate results. First, this study included only six EEG electrodes per subject compared to the 19 electrodes in the prior study. Second, this study used a wider age range (10 months to 37 years) than our prior work (4–11 years). Sleep spindle quantity and duration are affected by age (Campos‐Beltran & Marshall, 2021), thus the broader age range used in this study may have introduced variability. It is possible that sleep spindles are a reliable AS biomarker in a narrower developmental window than tested here. Third, it is possible that spindle quality during overnight sleep (as in this study) differs from spindle quality during the first ~20 min of sleep (as in our prior study). We addressed this possibility directly by analyzing spindles during the first 20 min of overnight sleep. This approach did not reveal any changes in spindle quantity in AS EEGs (Figure S6D); however, it did reveal a decrease in spindle duration in AS EEGs (Figure S6E). Fourth, this study (*n* = 12, three comparison groups) is under‐powered relative to our prior study (*n* = 13 for AS, *n* = 54 for NT, only two groups). Lastly, the presence of obstructive sleep apnea in a majority of subjects in this study could explain why spindle impairments were not reliably detected in AS. Prior work suggests that neurotypical children with obstructive sleep apnea have decreased spindle density relative to controls with no sleep apnea (Brockmann et al., 2018). However, the severity of sleep apnea was not correlated with sleep spindle quantity in our study (Figure S7). Thus, sleep apnea is unlikely to be the primary reason why spindle impairments were not reliably detected.

Use of a secondary spindle detector (YASA) did not dramatically improve our ability to detect impairments in AS EEGs, though we do report a trend toward fewer spindles (Figure 5g) and a statistically significant increase in peak spindle frequency in AS (Figure 5i). Subtle differences between spindle detectors must be considered for future study of sleep spindles in AS EEGs. While YASA is likely more accurate than the Kim/den Bakker detector when using neurotypical EEGs (Lacourse et al., 2019), one of its three detection parameters (relative sigma power) may not be well suited for AS EEGs. Relative sigma power is affected by relative power in other bands, because the total power must add up to 100%. Thus, increased delta power in AS EEGs may artificially depress the relative sigma signal, resulting in fewer detected spindles. More broadly, if sleep spindles are to be considered as an AS biomarker, optimizing the detection algorithm for AS EEGs would be one way to improve the likelihood of reliably detecting impairments.

The goal of this study was to evaluate EEG biomarkers for Angelman syndrome during overnight sleep. Overall, we conclude that overnight sleep is not necessary for detecting Angelman syndrome EEG biomarkers. Delta power was robustly and reliably increased in AS EEGs, especially earlier in development, but not more so during sleep. Sleep spindle impairments were less reliably detected than abnormal delta power. Given the challenges associated with performing overnight EEG studies in children with AS, it is encouraging that wake EEGs are sufficient, and perhaps ideal, for detecting delta in a clinical trial setting.

## CONFLICT OF INTEREST

Michael S. Sidorov has received consulting fees from Medpace, Inc. for EEG analysis.

## ETHIC STATEMENT

All studies were performed with approval of the UNC institutional review board (IRB # 18‐1729).

## Supporting information


**Data S1.**. Supporting information.

## Data Availability

The data that support the findings of this study are available from the corresponding author upon reasonable request.
