# Supplementary material for: Evaluation of electroencephalography biomarkers for Angelman syndrome during overnight sleep
Source: Autism Res. 2022 Mar 19;15(6):1031–42. doi: 10.1002/aur.2709 (PMC9227959; doi:10.1002/aur.2709)
Supplement: Supplementary file 1 — Data S1.. Supporting information. [file AUR-15-1031-s001.pdf]

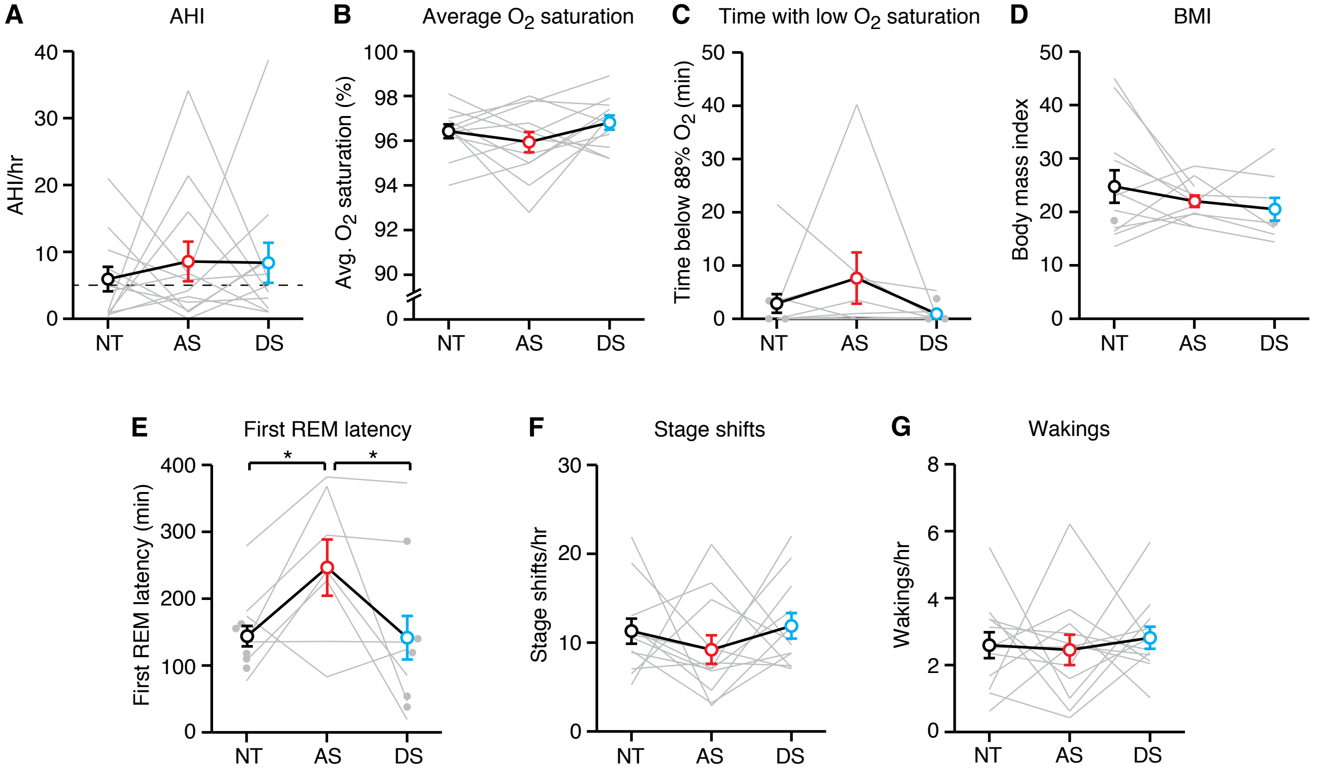

Supplementary Figure 1. Additional sleep quality assessments during overnight polysomnography. Neurotypical = NT, Angelman syndrome = AS, Down syndrome = DS. (A) Apnea/hypopnea index (AHI), a measure of the severity of sleep apnea, was similar between groups (RM ANOVA: $F_{(2,22)} = 0.2557$, $p = 0.7766$; $n = 12$ per group). Dotted line indicates AHI = 5, the typical cutoff for sleep apnea. (B) Average O_2 saturation throughout the night was similar by group (RM ANOVA: $F_{(2,22)} = 1.572$, $p = 0.2301$; $n = 12$ per group). (C) Time with low (below 88%) O_2 saturation was similar by group (mixed-effects analysis: $F_{(2,18)} = 1.862$, $p = 0.1841$; NT: $n = 12$, AS: $n = 8$, DS: $n = 12$). (D) Body mass index (BMI) was similar between groups (mixed-effects analysis: $F_{(2,17)} = 0.9556$, $p = 0.4043$; NT: $n = 12$, AS: $n = 11$, DS: $n = 7$). (E) Latency to first REM period during sleep was increased in AS (mixed-effects analysis: $F_{(2,17)} = 4.822$, $^*p = 0.0219$, post hoc NT-AS: $^*p = 0.0317$, post hoc AS-DS: $^*p = 0.0277$; NT: $n = 12$, AS: $n = 7$, DS: $n = 12$). (F) Stage shifts were similar between groups (RM ANOVA: main effect of genotype: $F_{(2,22)} = 0.7177$, $p = 0.4989$; $n = 12$ per group). (G) Wakings were similar between groups (RM ANOVA: main effect of genotype: $F_{(2,22)} = 0.1878$, $p = 0.8301$; $n = 12$ per group). Error bars indicate ± SEM.

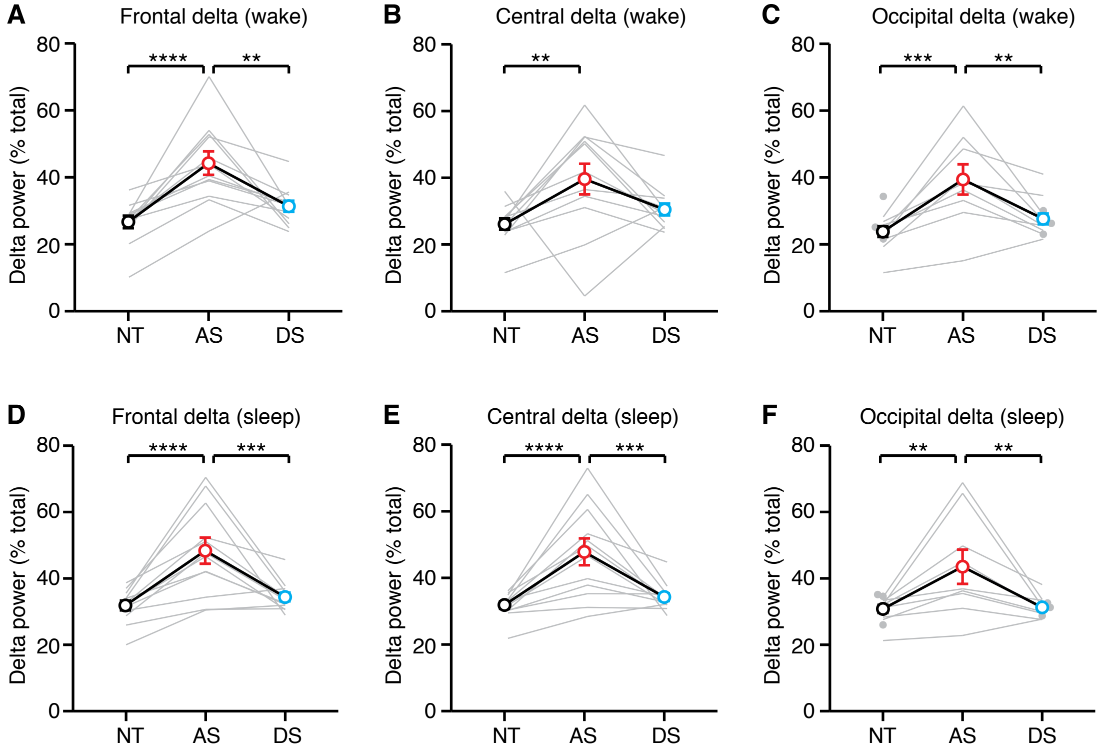

Supplementary Figure 2. Delta power is increased in Angelman syndrome across all EEG electrodes. Neurotypical = NT, Angelman syndrome = AS, Down syndrome = DS. (A-C) Delta by region during wake. (A) Frontal delta is increased in AS during wake (RM ANOVA: $F_{(2,22)} = 15.77$, ****$p < 0.0001$; post hoc NT-AS: ****$p < 0.0001$; post hoc AS-DS: **$p = 0.0018$; $n = 12$ per group). (B) Central delta is increased in AS during wake (RM ANOVA: $F_{(2,22)} = 5.715$, *$p = 0.0100$; post hoc NT-AS: **$p = 0.0085$; post hoc AS-DS: $p = 0.0875$; $n = 12$). (C) Occipital delta is increased in AS during wake (mixed effects analysis: $F_{(2,19)} = 14.39$, ***$p = 0.0002$; post hoc NT-AS: ***$p = 0.0001$; post hoc AS-DS: **$p = 0.0022$; WT: $n = 12$, AS: $n = 9$, DS: $n = 12$). (D-F) Delta by region during sleep. (D) Frontal delta is increased in AS during sleep (RM ANOVA: $F_{(2,22)} = 17.78$, ****$p < 0.0001$; post hoc NT-AS: ****$p < 0.0001$; post hoc AS-DS: ***$p = 0.0003$; $n = 12$). (E) Central delta is increased in AS during sleep (RM ANOVA: $F_{(2,22)} = 15.99$, ****$p < 0.0001$; post hoc NT-AS: ****$p < 0.0001$; post hoc AS-DS: ***$p = 0.0006$; $n = 12$). (F) Occipital delta is increased in AS during sleep (mixed effects analysis: $F_{(2,19)} = 9.527$, **$p = 0.0014$; post hoc NT-AS: **$p = 0.0023$; post hoc AS-DS: **$p = 0.0032$; WT: $n = 12$, AS: $n = 9$, DS: $n = 12$). Error bars indicate ± SEM.

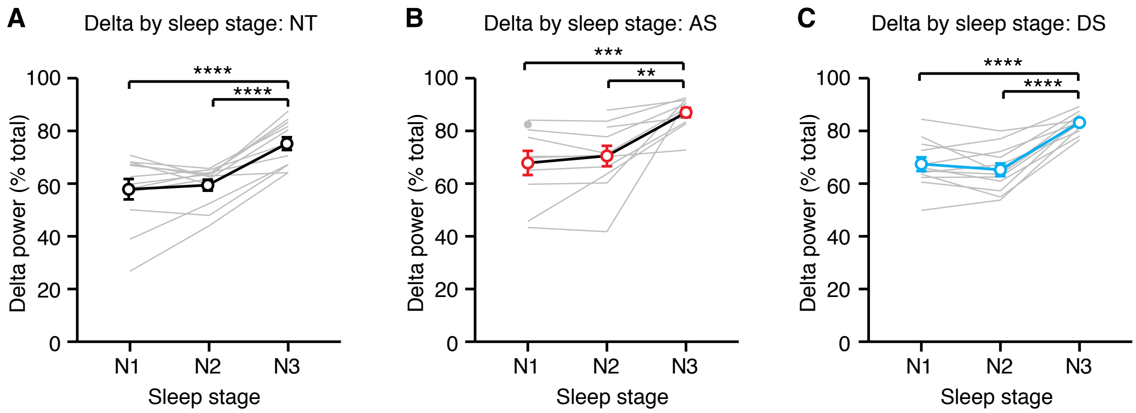

Supplementary Figure 3. Delta power is greater in all genotypes during stage N3 of sleep. Neurotypical = NT, Angelman syndrome = AS, Down syndrome = DS. (A) Delta (defined here as 1-4 Hz) is increased in N3 relative to N1 and N2 in NT EEGs (RM ANOVA: $F_{(2,22)} = 30.01$, $p < 0.0001$; $n = 12$ per group; **** indicates $p < 0.0001$ on post hoc tests). (B) Delta is increased in N3 relative to N1 and N2 in AS EEGs (mixed-effects analysis: $F_{(2,18)} = 13.91$, $p = 0.0002$; NT: $n = 10$, AS: $n = 11$, DS: $n = 11$; ** indicates $p < 0.01$ and *** indicates $p < 0.001$ on post hoc tests). (C) Delta is increased in N3 relative to N1 and N2 in DS EEGs (RM ANOVA: $F_{(2,22)} = 47.34$, $p < 0.0001$; $n = 12$ per group; **** indicates $p < 0.0001$ on post hoc tests).

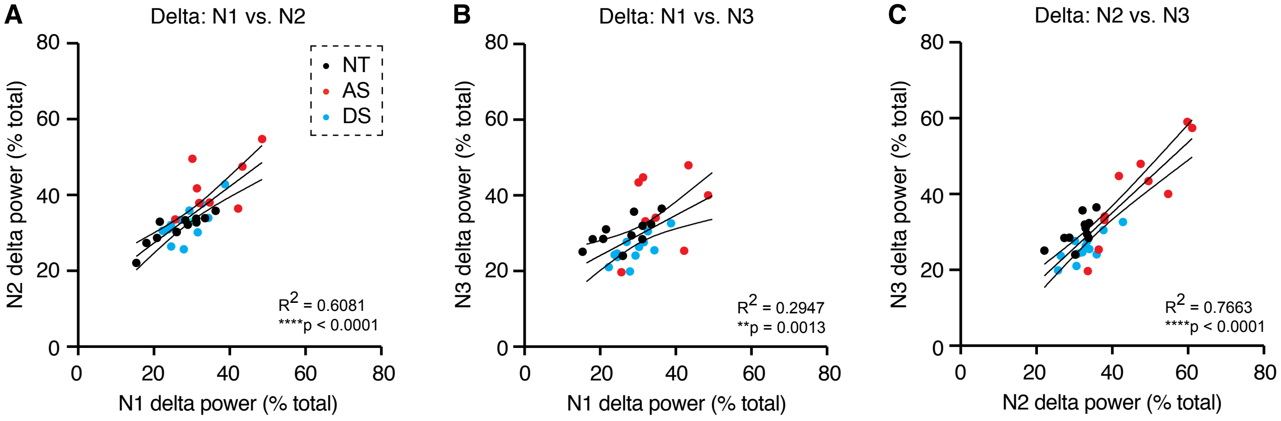

Supplementary Figure 4. Delta power is correlated between sleep stages within individuals. Neurotypical = NT, Angelman syndrome = AS, Down syndrome = DS. (A) Delta power during N1 and N2 are positively correlated ($R^2 = 0.6239$, ****$p < 0.0001$). (B) Delta power during N1 and N3 are positively correlated ($R^2 = 0.3179$, ***$p < 0.0006$). (C) Delta power during N2 and N3 are positively correlated ($R^2 = 0.7722$, ****$p < 0.0001$). Overlaid lines represent best-fit ± 95% confidence interval.

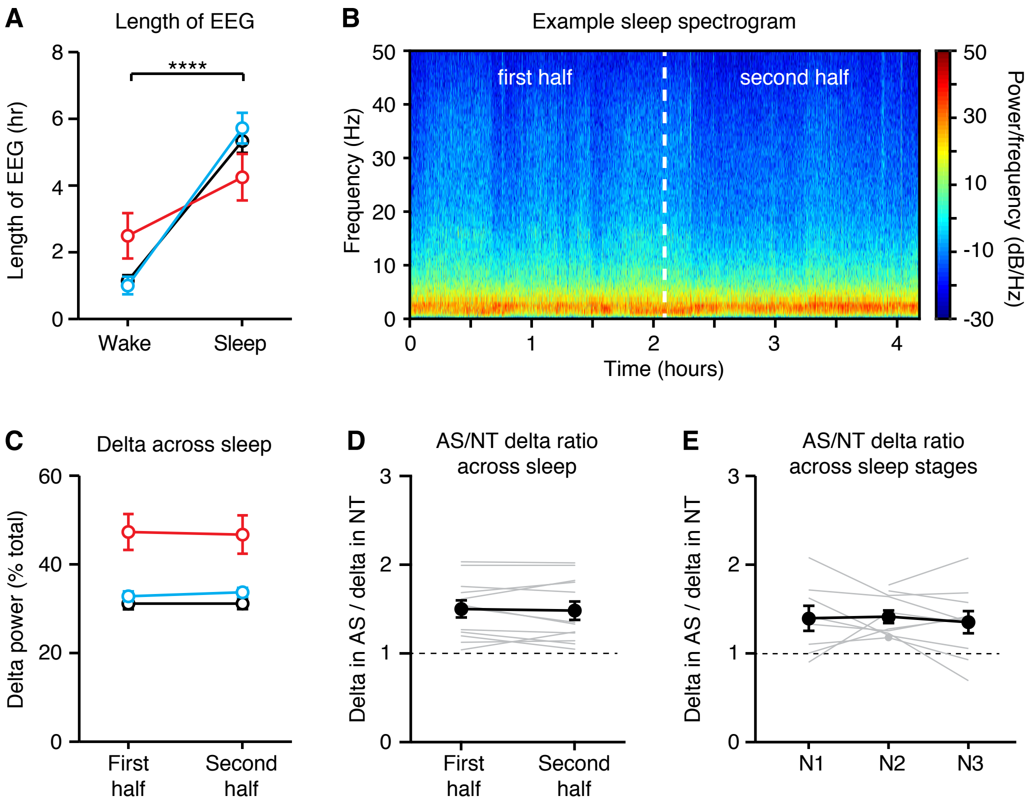

Supplementary Figure 5. Delta power is consistent across the duration of sleep EEGs. (A) Sleep periods of EEG were significantly longer than wake periods of EEG (two-way RM ANOVA: main effect of sleep/wake state: $F_{(1,11)} = 37.67$, ****$p < 0.0001$; $n = 12$ per group). (B) Example spectrogram from an AS sleep EEG (channel F3 in 2 year old boy; concatenated with periods of wake and artifacts removed), illustrating the stability of delta over time. (C) Quantification reveals no difference in delta power between the first and second half of NREM sleep EEGs (two-way RM ANOVA: main effect of time: $F_{(1,11)} = 0.06227$, $p = 0.8075$; $n = 12$). (D) AS/NT delta ratio is consistent across the first and second half of NREM sleep (paired t-test; $t_{(11)} = 0.4861$, $p = 0.6364$; $n = 12$). (E) AS/NT delta ratio is consistent across sleep stages (RM ANOVA: $F_{(2,16)} = 0.4971$, $p = 0.6174$; N1: $n = 8$, N2: $n = 11$, N3: $n = 10$). Error bars indicate ± SEM. Dotted line indicates an AS/NT delta ratio of "1". Values greater than 1 indicate pairs where the AS EEG has greater delta power than its paired NT EEG.

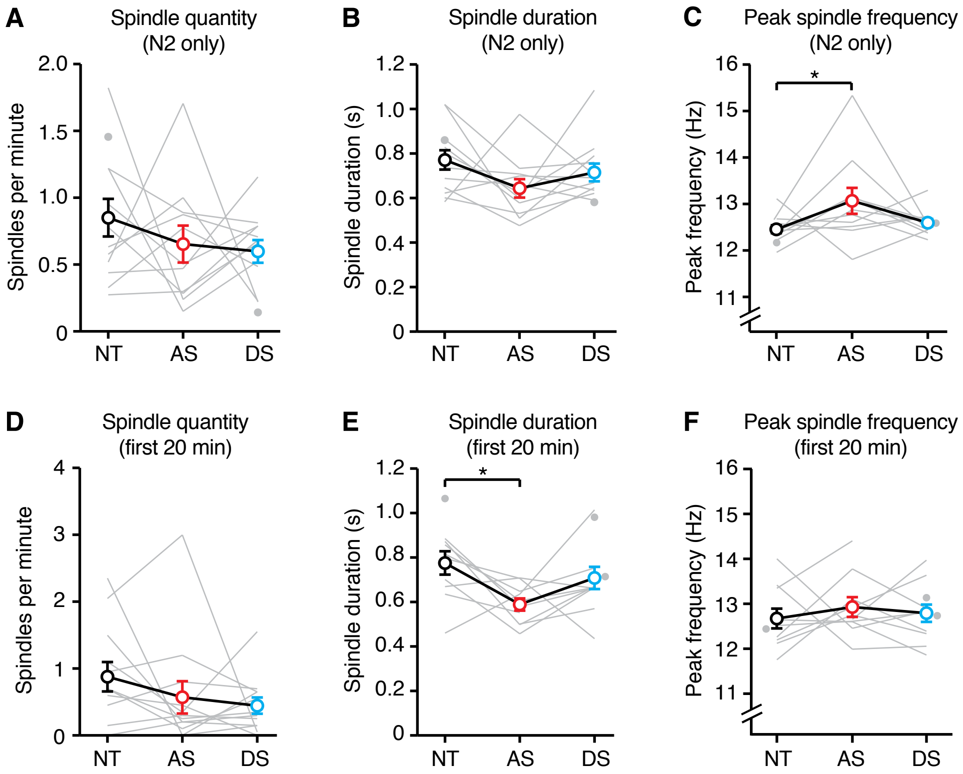

Supplementary Figure 6. Spindle detection during stage N2 and the first twenty minutes of NREM sleep. Neurotypical = NT, Angelman syndrome = AS, Down syndrome = DS. (A-C) Spindle detection during stage N2 using the Kim/den Bakker detector. (A) Spindle quantity during N2 is not different by group (mixed-effects analysis: $F_{(2,32)} = 1.185$, $p = 0.3188$; NT: $n = 12$, AS: $n = 11$, DS: $n = 12$). (B) Spindle duration during N2 is not different by group (mixed-effects analysis: $F_{(2,21)} = 2.396$, $p = 0.1156$; $n = 12$, AS: $n = 11$, DS: $n = 12$). (C) Peak spindle frequency during N2 is higher in AS EEGs (mixed-effects analysis: $F_{(2,32)} = 3.575$, $*p = 0.0397$; post hoc NT-AS: $*p = 0.0390$; post-hoc AS-DS: $p = 0.1352$; $n = 12$, AS: $n = 11$, DS: $n = 12$). (D-F) Spindle detection during the first 20 minutes of NREM sleep using the Kim/den Bakker detector. (D) Spindle quantity is not different by group (RM ANOVA: $F_{(2,32)} = 3.575$, $*p = 0.0397$; $n = 12$). (E) Spindle duration is decreased in AS (mixed-effects analysis: $F_{(2,28)} = 4.229$, $*p = 0.0248$; post hoc NT-AS: $*p = 0.0205$; post hoc AS-DS: $p = 0.1636$; NT: $n = 10$, AS: $n = 10$, DS: $n = 11$). (F) Peak spindle frequency is not different by group (mixed-effects analysis: $F_{(2,28)} = 0.3626$, $p = 0.6991$; NT: $n = 10$, AS: $n = 10$, DS: $n = 11$).

Peak spindle frequency is not statistically different between groups ($F_{(2,22)} = 2.852$, $p = 0.0792$).

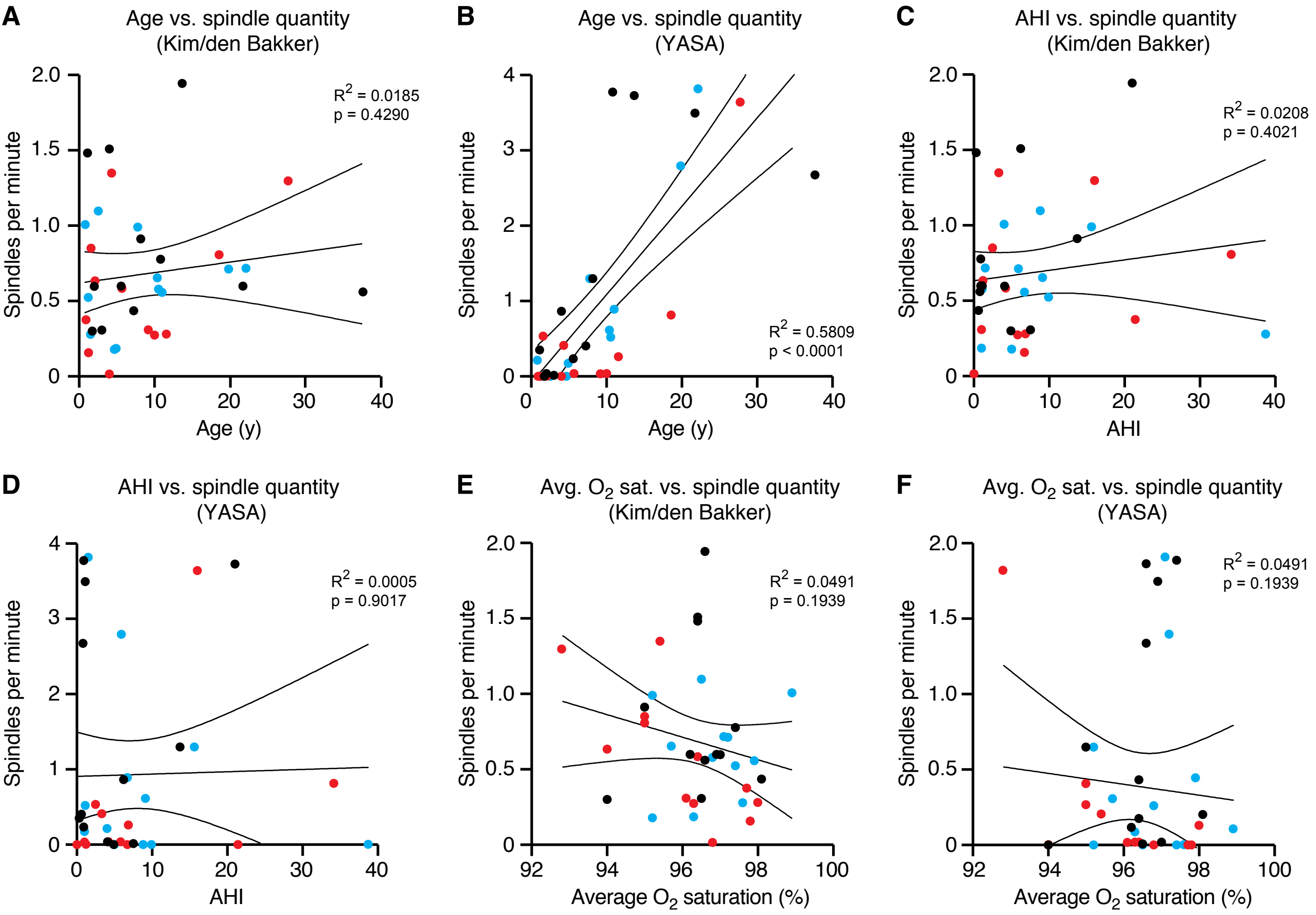

Supplementary Figure 7. Relationships between spindles, age, and sleep apnea. Black: NT; red: AS; blue: DS. (A) Sleep spindle quantity, as detected by the Kim/den Bakker detector, is not significantly correlated with age. (B) Sleep spindle quantity, as detected by YASA, is correlated with age. (C-D) AHI and (E-F) average oxygen saturation are not correlated with sleep spindle quantity.
